# ATM protein is deficient in over 40% of lung adenocarcinomas

**DOI:** 10.18632/oncotarget.9757

**Published:** 2016-06-01

**Authors:** Liza C. Villaruz, Helen Jones, Sanja Dacic, Shira Abberbock, Brenda F. Kurland, Laura P. Stabile, Jill M. Siegfried, Thomas P. Conrads, Neil R. Smith, Mark J. O'Connor, Andrew J. Pierce, Christopher J. Bakkenist

**Affiliations:** ^1^ University of Pittsburgh Cancer Institute, University of Pittsburgh School of Medicine, Pittsburgh, PA, USA; ^2^ Astrazeneca, Cambridge, United Kingdom; ^3^ Department of Pathology, University of Pittsburgh School of Medicine, Pittsburgh, PA, USA; ^4^ Department of Biostatistics, University of Pittsburgh School of Public Health, Pittsburgh, PA, USA; ^5^ Department of Pharmacology and Chemical Biology, University of Pittsburgh School of Medicine, Pittsburgh, PA, USA; ^6^ Department of Pharmacology, University of Minnesota, Minneapolis, MN, USA; ^7^ Inova Schar Cancer Institute, Inova Center for Personalized Health, Falls Church, VA, USA; ^8^ Department of Radiation Oncology, University of Pittsburgh School of Medicine, Pittsburgh, PA, USA

**Keywords:** ataxia telangiectasia mutated (ATM), ATM and Rad-3-related (ATR), lung adenocarcinoma, non-small cell lung cancer (NSCLC)

## Abstract

Lung cancer is the leading cause of cancer-related mortality in the USA and worldwide, and of the estimated 1.2 million new cases of lung cancer diagnosed every year, over 30% are lung adenocarcinomas. The backbone of 1^st^-line systemic therapy in the metastatic setting, in the absence of an actionable oncogenic driver, is platinum-based chemotherapy. ATM and ATR are DNA damage signaling kinases activated at DNA double-strand breaks (DSBs) and stalled and collapsed replication forks, respectively. ATM protein is lost in a number of cancer cell lines and ATR kinase inhibitors synergize with cisplatin to resolve xenograft models of ATM-deficient lung cancer. We therefore sought to determine the frequency of ATM loss in a tissue microarray (TMA) of lung adenocarcinoma. Here we report the validation of a commercial antibody (ab32420) for the identification of ATM by immunohistochemistry and estimate that 61 of 147 (41%, 95% CI 34%-50%) cases of lung adenocarcinoma are negative for ATM protein expression. As a positive control for ATM staining, nuclear ATM protein was identified in stroma and immune infiltrate in all evaluable cases. ATM loss in lung adenocarcinoma was not associated with overall survival. However, our preclinical findings in ATM-deficient cell lines suggest that ATM could be a predictive biomarker for synergy of an ATR kinase inhibitor with standard-of-care cisplatin. This could improve clinical outcome in 100,000's of patients with ATM-deficient lung adenocarcinoma every year.

## INTRODUCTION

ATM (ataxia telangiectasia-mutated) and ATR (ataxia telangiectasia and Rad 3-related) are serine/threonine protein kinases that phosphorylate a broad and overlapping catalogue of several thousand substrates that collectively impact cell cycle progression, DNA replication and repair, transcription, translation and metabolism [1–3]. Above poorly defined thresholds of DNA damage, ATM and ATR kinase signaling can also lead to either apoptosis or senescence [1–3]. ATM is activated within seconds to minutes of exposure to ionizing radiation (IR) and other agents that induce DNA double-strand breaks (DSBs) [4–7]. ATR is activated within minutes to hours of exposure to agents that induce stalled and collapsed replication forks and at DSBs after end-resection [8].

Mutations in ATM that result in the absence of detectable protein cause the childhood disease ataxia telangiectasia (A-T) [4, 9]. A-T is characterized by progressive neurodegeneration, increased incidence of lymphoid malignancies and profound radiosensitivity [4, 9, 10]. Mice that express no ATM protein are viable, have an increased incidence of lymphoid malignancies and are radiosensitive, but show no signs of neurodegeneration [11, 12]. In contrast, expression of ATM kinase-inactive protein causes early embryonic lethality in mice [13, 14]. Deletion of ATR also causes early embryonic lethality in mice [15]. Thus, ATM loss may allow tumor cells a proliferation and survival advantage, while mutations that inactivate ATM kinase and ATR loss may be lethal. Acquired ATM loss may be exploited for clinical benefit as pharmacologic ATR kinase inhibitors including VE-821, VE-822/VX970 and AZD6738 synergize with cisplatin to kill ATM-deficient cancer cells *in vitro* and to resolve xenograft models of ATM-deficient lung cancer *in vivo* [16–19].

The frequency of loss of ATM protein has not been reported in lung cancer previously. In large-scale genomics efforts, fourteen mutations, including 1 nonsense, 1 splice-site and 2 frameshift mutations, were identified in 13 of 188 (7%) lung adenocarcinomas [20]. Twenty-two mutations, including 2 nonsense, 1 splice-site and 1 frameshift mutation, were identified in 20 of 183 (11%) lung adenocarcinomas [21]. Molecular profiling identified 25 mutations, including 8 nonsense, 1 splice site and 4 frame-shift mutations, in 24 of 230 (10%) lung adenocarcinomas, and amplification and deletion were each identified in 3 lung adenocarcinomas [22]. In total, in the latter study, ATM was altered in 27 of 230 (12%) lung adenocarcinomas. It is important to consider that the majority of somatic mutations identified in ATM have no known functional consequence. Here we report the validation of a commercial antibody for the detection of ATM by immunohistochemistry. Remarkably we show that 61 of 149 (41%) lung adenocarcinomas can be considered negative for ATM protein expression. This finding is important as the combination of an ATR kinase inhibitor with standard-of-care cisplatin may improve clinical outcome in 100,000's of patients with ATM-deficient lung adenocarcinoma every year.

## RESULTS

### Selectivity of anti-ATM antibody [Y170] (ab32420)

**Figure 1:**
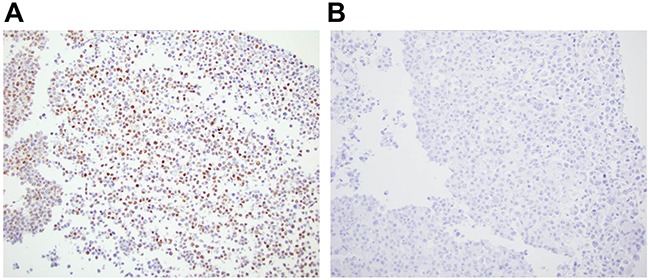
Rabbit monoclonal anti-ATM antibody [Y170] (ab32420) identifies a nuclear antigen in human lymphoblastoid cells expressing ATM **A.** GM14680 cells that express ATM, and **B.** GM01526 cells derived from an ataxia telangiectasia patient that express no ATM detectable protein by immunoblotting.

**Figure 2:**
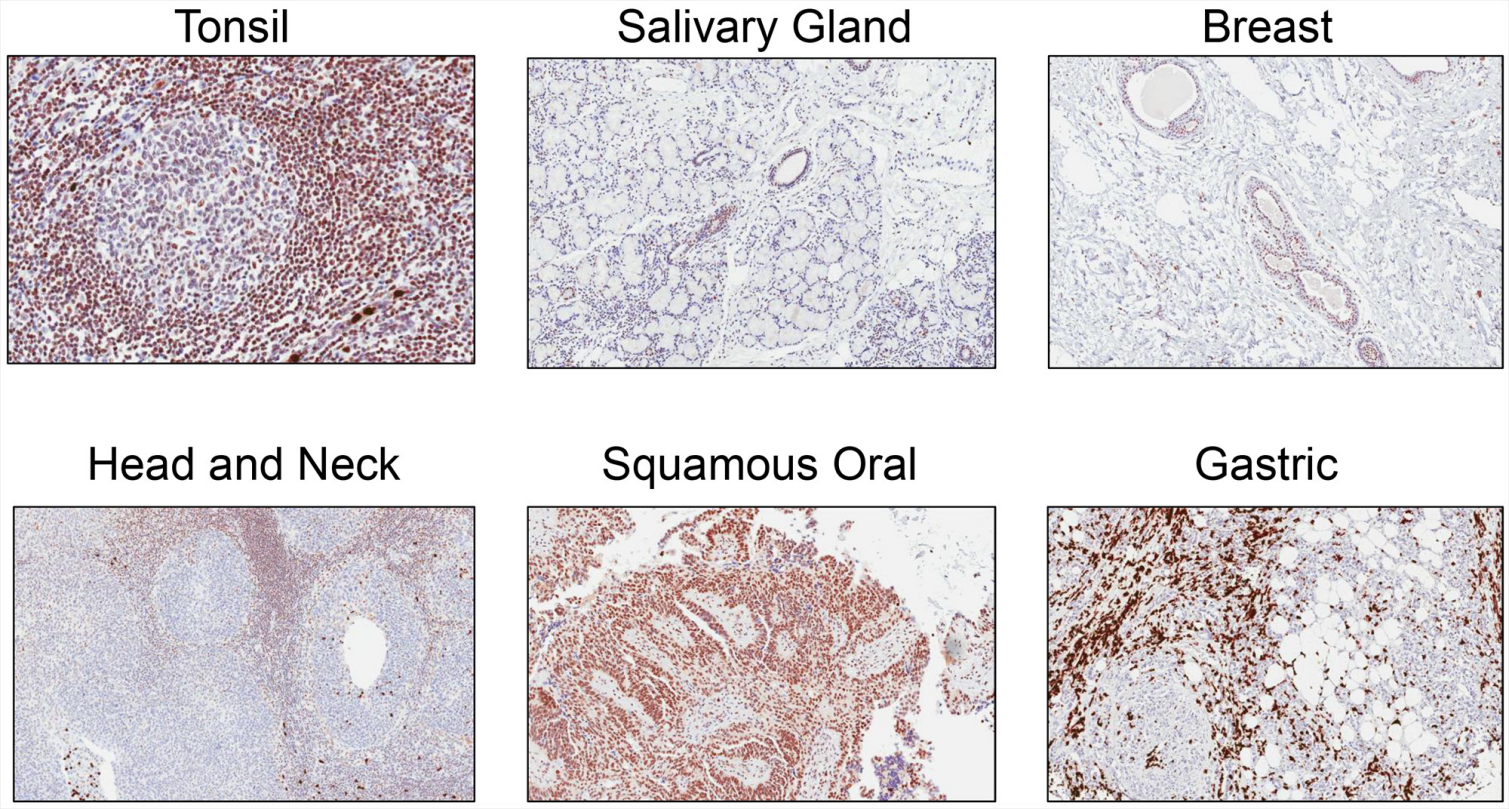
Rabbit monoclonal anti-ATM antibody [Y170] (ab32420) identifies a nuclear antigen in formalin fixed, paraffin embedded human cancers Ab32420 identified a nuclear antigen in a tonsil, salivary gland, breast, head and neck cancer, squamous oral cancer and gastric cancer.

**Figure 3:**
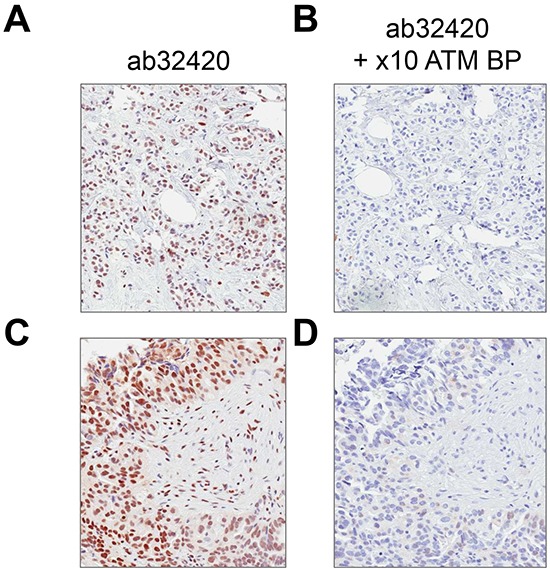
Rabbit monoclonal anti-ATM antibody [Y170] (ab32420) staining is blocked by immunizing peptide **A/C.** Ab32420 positive control with no blocking peptide, **B/D.** ab32420 incubated with a 10 × excess of immunizing peptide at 4°C overnight.

**Figure 4:**
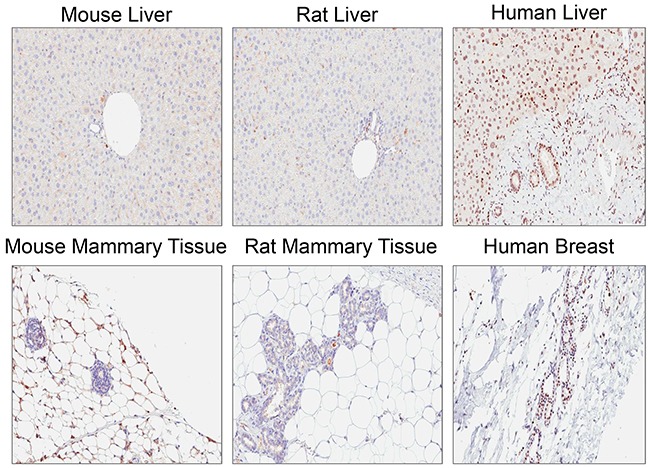
Rabbit monoclonal anti-ATM antibody [Y170] (ab32420) does not detect antigen in mouse and rat

Rabbit monoclonal anti-ATM antibody [Y170] (ab32420) identified a nuclear antigen in tissue sections generated from formalin fixed, paraffin embedded (FFPE) human lymphoblastoid cells (GM14680) that express ATM, but not in lymphoblastoid cells (GM01526) derived from an ataxia telangiectasia patient that express no detectable protein by immunoblotting (Figure 1). Basic immunohistochemistry controls including no primary antibody and isotype control primary antibody were negative for staining (Supplementary Figures S1 and S2). Ab32420 also identified a nuclear antigen in a tonsil, salivary gland, breast, head and neck cancer, squamous oral cancer and gastric cancer (Figure 2). Positive staining was reversed by prior incubation of ab32420 with the peptide antigen used to generate the antibody (Figure 3). Ab32420 did not detect antigen in tissue sections generated from FFPE mouse and rat liver or mammary tissue (Figure 4). Rabbit monoclonal anti-ATM antibody ab32420 was generated using a synthetic peptide identical to the human ATM protein around serine-1981. The human ATM sequence around human serine 1981 is EKRSLAFEEGSQSTTISSLSE; the mouse ATM sequence around serine 1987 is EKRSPTFEEGSQGTTISSLSE; the rat ATM sequence around serine 1987 is EKRSPTFEEGSQGTTISSLSE. Thus, ab32420 recognizes a human-specific antigen in immunohistochemistry whose expression is consistent with that of the ATM protein. Since serine-1981 is phosphorylated after IR and exposure to other agents that induce DSBs [7, 23, 24], and also in human cancers as a consequence of replicative stress [25, 26], and because phosphorylation on ATM serine-1981 can have a very high stoichiometry [7], it is important to determine whether ab32420 identifies either the non-phosphorylated or phosphorylated protein selectively.

**Figure 5:**
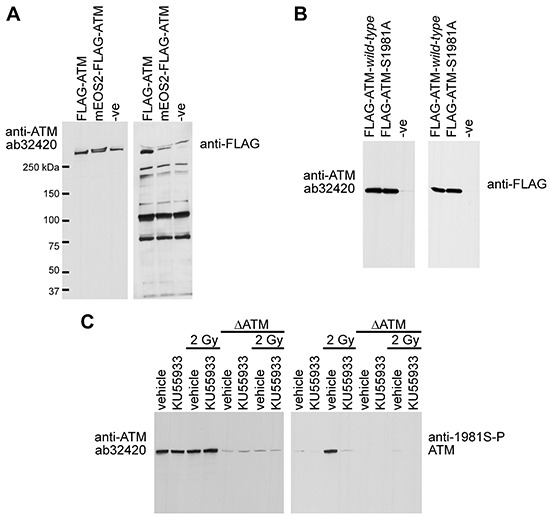
Rabbit monoclonal anti-ATM ab32420 does not selectively identify either the non-phosphorylated or phosphorylated form of ATM in immunoblotting **A.** FLAG-ATM and mEOS2-FLAG-ATM were expressed in 293T cells and whole cell lysates were immunoblotted with either ab32420 or anti-FLAG M5 antibody. **B.** FLAG-ATM *wild-type* and FLAG-ATM serine-1981-alanine mutant were expressed in 293T cells and recombinant proteins were immunoprecipitated using anti-FLAG M2 agarose and immunoblotted with either ab32420 or anti-FLAG M5. **C.** H460 lung adenocarcinoma cells stably expressing either an shRNA that targets ATM or a scrambled shRNA were treated with KU55933, a small molecule ATM kinase inhibitor and exposed to 2 Gy IR. Whole cell extracts were generated, resolved and immunoblotted with either ab32420 or anti-FLAG M5.

To determine the phospho-selectivity of ab32420 in immunoblotting, FLAG-epitope tagged ATM (FLAG-ATM) and fluorescent protein tagged ATM (mEOS2-FLAG-ATM) were expressed in human embryonic kidney 293T cells and whole cell lysates were prepared [14, 23]. Whole cell lysates were resolved and immunoblotted with either ab32420 or anti-FLAG M5 antibody (Figure 5A). Ab32420 and anti-FLAG identified a single band in the extract expressing FLAG-ATM as endogenous ATM and FLAG-ATM are not resolved by SDS-PAGE. Ab32420 and anti-FLAG identified two bands in the extract expressing mEOS2-FLAG-ATM as endogenous ATM and mEOS2-FLAG-ATM are resolved by SDS-PAGE. The anti-FLAG antibody identifies the N-terminal FLAG-tagged ATM protein more efficiently than the internal FLAG-tagged mEOS2-FLAG-ATM as expected. Ab32420, but not anti-FLAG, identified a single band in the whole cell extract generated from mock-transfected cells.

To determine whether ATM serine-1981 is essential for ab32420 binding, FLAG-ATM *wild-type* and FLAG-ATM serine-1981-alanine mutant were expressed in 293T cells and recombinant proteins were immunoprecipitated using anti-FLAG M2 agarose [7]. Immunoprecipitates were resolved and immunoblotted with either ab32420 or anti-FLAG M5. Ab32420 identified both FLAG-ATM *wild-type* and FLAG-ATM serine-1981-alanine mutant (Figure 5B). Since ATM detection with ab32320 and anti-FLAG are comparable, substitution of serine-1981 for alanine-1981 does not compromise detection of ATM by ab32420.

To determine whether ATM serine-1981 phosphorylation disrupts ATM identification by ab32420, H460 lung adenocarcinoma cells stably expressing either an shRNA that targets ATM or a scrambled shRNA [27], were treated with KU55933, a small molecule ATM kinase inhibitor [28], and exposed to 2 Gy IR. Whole cell extracts were generated, resolved and immunoblotted with either ab32420 or anti-FLAG M5 (Figure 5C). Neither the radiation nor the ATM kinase inhibitor affected the identification of ATM by ab32420 even though serine-1981 was efficiently phosphorylated by radiation treatment in the absence of an ATM inhibitor. We conclude that ab32420 does not selectively identify either the non-phosphorylated or phosphorylated form of ATM in immunoblotting, but rather recognizes total ATM protein in western blotting.

**Figure 6:**
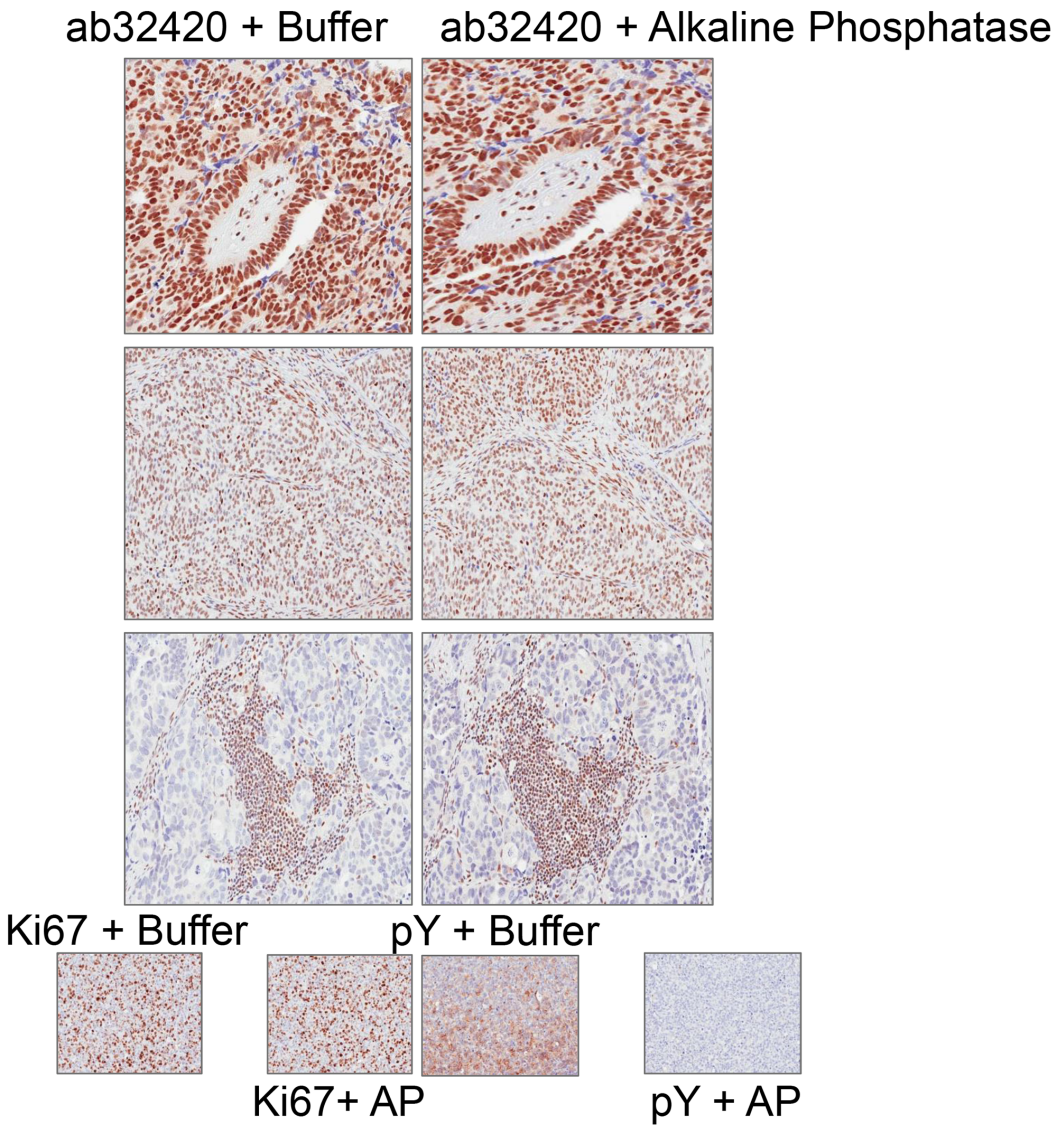
Rabbit monoclonal anti-ATM ab32420 does not selectively identify either the non-phosphorylated or phosphorylated form of ATM in immunohistochemistry ab32420 and Ki67 staining is not affected by alkaline phosphatase treatment of tissue sections. Phosphotyrosine (pY) staining is lost after alkaline phosphatase treatment of tissue sections.

To determine whether phosphorylation disrupts epitope identification by ab32420 in immunohistochemistry (IHC), tissue sections were incubated with alkaline phosphatase prior to incubation with ab32420. Alkaline phosphatase treatment did not change the identification of the nuclear antigen by ab32420 (Figure 6). We conclude that there is no selectivity of ab32420 for the non-phosphorylated or phosphorylated form of ATM in IHC. These controls are important since the stoichiometry of ATM phosphorylation in primary fibroblasts is at least 50% following 2 Gy IR [7], and ATM serine-1981 phosphorylation would result in false negative/reduced staining for ATM if the phosphorylated protein were not identified by ab32420.

### ATM expression in a lung adenocarcinoma TMA

**Figure 7:**
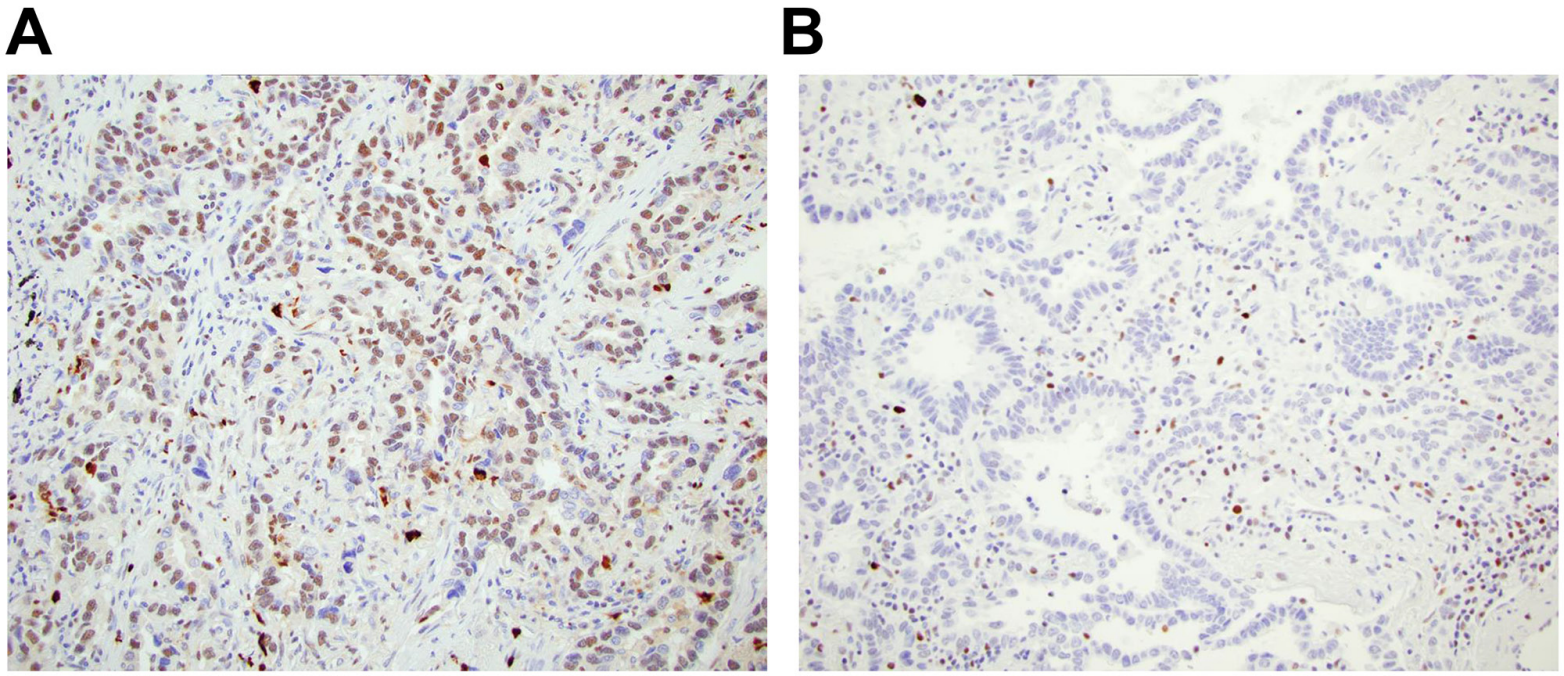
ATM loss is identified in lung adenocarcinoma **A.** ATM positive lung adenocarcinoma, and **B.** ATM loss in lung adenocarcinoma with ATM positive staining in immune infiltrate.

A tissue microarray (TMA) of lung adenocarcinomas obtained from the the University of Pittsburgh Lung Specialized Program of Research Excellence (SPORE) was stained for ATM at the Biomarker Division of AstraZeneca in Cambridge, UK. An immortalized cell line from an ataxia telangiectasia individual that expresses no ATM and a similar cell line from a patient that expressed ATM were used as technical controls (Figure 1). Unambiguous positive and negative nuclear staining was identified by ab32420 in the lung adenocarcinomas (Figure 7).

Patient tumors were considered evaluable if at least two cores were stained for ATM in the stroma and immune infiltrate, since ATM is known to be expressed at high level in lymphocytes [24]. Of the 227 unique patients in the TMA, 147 had evaluable lung adenocarcinomas (Table 1), with most exclusions due to inadequate tissue or negative staining in the lymphocytes or stroma. A core was scored as ATM negative if >90% of tumor cells were negative for nuclear ATM staining and <10% of tumor cells were positive for ATM staining, as previously described [29, 30]. If any of 2 or more cores stained positive for ATM in tumor cell nuclei that case was scored as positive. The distribution of ATM staining in tumor cores tends to follow a bi-modal distribution with clear distinction between “positive” and “negative” staining cores. The 90% negative tumor nuclei cutoff for ATM low/negative used here was chosen to be consistent with that previously established in the literature [30]. Overall, 61 (41%, 95% confidence interval 34%-50%) of lung adenocarcinomas were ATM negative and 86 (59%, 95% confidence interval 50%-66%) were ATM positive.

**Table 1:** ATM loss is not associated with clinical pathologic features of lung adenocarcinomas

	All (n=147)	ATM Negative (n=61)	ATM Positive (n=86)	P-value
	N (%)	N (%)	N (%)	
Age, mean (range)	65 (39-88)	66 (44-88)	65 (39-87)	0.43^b^
Gender				0.74^c^
Female	78 (53)	31 (51)	47 (55)	
Male	69 (47)	30 (49)	39 (45)	
Race				0.86^c^
White	134 (91)	55 (90)	79 (92)	
Black	8 (6)	4 (6)	4 (5)	
Pacific Islander	2 (1)	1 (2)	1 (1)	
Unknown	3 (2)	1 (2)	2 (2)	
Genotype				0.20^d^
EGFR	39 (26)	18 (30)	21 (24)	
KRAS	73 (50)	33 (54)	40 (47)	
WT	35 (24)	10 (16)	25 (29)	
Smoking History				0.53^d^
Current	40 (27)	15 (25)	25 (29)	
Former	87 (59)	35 (57)	52 (60)	
Never	19 (13)	10 (16)	9 (10)	
Unknown	1 (1)	1 (2)		
Pathologic Stage (AJCC version 7)				0.64^b^
IA	40 (27)	16 (26)	24 (28)	
IB	33 (22)	13 (21)	20 (23)	
IIA-IIB	37 (25)	14 (23)	23 (27)	
IIIA-IIIB	26 (18)	13 (21)	13 (15)	
4	10 (7)	4 (7)	6 (7)	
N/A^a^	1 (1)	1 (2)		
Surgical Procedure				0.001^c^
Lobectomy	119 (81)	51 (84)	68 (79)	
Pneumonectomy	8 (5)	7 (11)	1 (1)	
Wedge Resection	20 (14)	3 (5)	17 (20)	
Adjuvant Chemotherapy				0.40^c^
No	142 (97)	60 (98)	82 (95)	
Yes	5 (3)	1 (2)	4 (5)	
Angiolymphatic invasion				0.30^d^
No	65 (44)	24 (39)	41 (48)	
Yes	79 (54)	36 (59)	43 (50)	
Unknown	3 (2)	1 (2)	2 (2)	
Pleural invasion				0.49^d^
No	87 (59)	38 (62)	49 (57)	
Yes	58 (39)	22 (36)	36 (42)	
Unknown	2 (1)	1 (2)	1 (1)	
Inflammation				0.60^d^
Mild	99 (67)	39 (64)	60 (70)	
Moderate	35 (24)	17 (28)	18 (21)	
Severe	11 (7)	4 (6)	7 (8)	
Unknown	2 (1)	1 (2)	1 (1)	

AJCC = American Joint Committee on Cancer

a No lymph nodes removed-cannot assess pathologic stage

b Wilcoxon rank sum test

c Fisher's exact test

d Chi-square test

**Figure 8:**
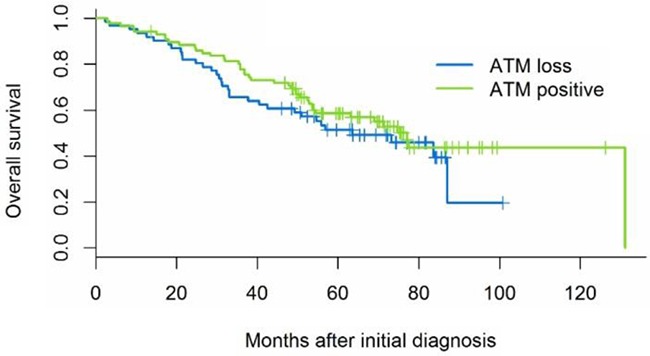
Kaplan-Meier survival curves illustrate that ATM is not associated with overall survival (log-rank p-value 0.33)

ATM deficiency was not associated with age, gender, race, EGFR or KRAS mutation, smoking history, pathologic stage, degree of inflammation, or presence of angiolymphatic or pleural invasion (Table 1). Overall survival following diagnosis ranged from 2-131 months (median 34 months) for the 74 patients (50%) with recorded date of death; the remaining 73 patients were known to be alive after 14-126 months of follow-up. ATM status was not associated with overall survival, either in univariate analysis (Figure 8, Table 2, log-rank p-value=0.33) or controlling for known prognostic factors pathologic stage (rank-order as continuous variable 1-7), age, sex, and genotype (hazard ratio for ATM positive 1.18, 95% CI 0.72-1.92, Wald p-value=0.52).

**Table 2:** Survival Analysis

	1 Year Overall Survival Rate (95% confidence interval)	5 Year Overall Survival Rate (95% confidence interval)	Median Overall Survival (95% confidence interval)
ATM Positive N=86	94% (87-98 months)	59% (47-68 months)	77 months (54-131 months)
ATM Negative N=61	93% (83-97 months)	51% (38-63 months)	64 months (40–NE months)

Overall survival landmarks did not differ by ATM status.

NE = not evaluable.

## DISCUSSION

We show here that 61 of 147 (41%) of lung adenocarcinomas have been demonstrated to be negative for ATM protein expression. In large-scale genomics efforts, ATM coding sequence alterations were identified in ≤12% lung adenocarcinoma [20–22]. Our validation experiments show that ab32420 recognizes human ATM and that there is no selectivity of ab32420 for either ATM serine-1981 or ATM phosphoserine-1981. There is no evidence for another post-translational modification within 8 amino acids of serine-1981 in ATM. Thus, the available data suggest that a yet to be identified mechanism controls either the translation or degradation of ATM protein in lung adenocarcinoma.

This is not the first report of rabbit monoclonal anti-ATM antibody ab32420 in IHC. Anti-ATM antibody ab32420 identified ATM reduction or loss in 158 of 314 (59%) of colorectal cancers and this was associated with worse disease-free survival [31]. Anti-ATM antibody ab32420 identified ATM loss in 67 of 555 (12%) of pancreatic cancers and patients with ATM loss and normal *TP53* had worse overall survival [32]. A second study identified loss of ATM phosphorylation on serine-1981 in 54 of 133 (41%) of pancreatic cancers and this also associated with worse overall survival [33]. Anti-ATM antibody ab32420 identified ATM loss or reduction in 616 of 1183 (52%) and ATM high in 567 of 1183 (48%) breast cancers and ATM loss or reduction was associated with poor survival in estrogen receptor negative breast cancer [34]. In a second study, anti-ATM antibody ab32420 identified ATM low in 22 of 168 (13%) and ATM high in 79 of 168 (47%) hormone negative breast cancer and ATM low in 36/130 (28%) and ATM high in 66/130 (51%) hormone positive breast cancers [35]. In this study, ATM low associated poorer survival outcomes and this effect was more pronounced in the hormone negative breast cancers where a multivariate analysis demonstrated that ATM low predicted survival independent of tumor size and lymph node status [35]. The assay described in detail here has also been used by AstraZeneca to select patients with recurrent or metastatic gastric cancer and ATM loss for a trial of olaparib plus paclitaxel followed by maintenance on olaparib [29]. Thus, IHC with ab32420 has clinical utility as a biomarker for prognosis and, potentially, personalized medicine.

Lung cancer cell lines are sensitized to cisplatin by ATR kinase inhibitors *in vitro* and in cell line and patient derived xenografts (PDX) *in vivo* [16, 17, 36, 37]. Furthermore, cisplatin and ATR kinase inhibitors synergize to kill ATM-deficient lung cancer cells *in vitro* and to resolve ATM-deficient xenografts *in vivo* [16–18]. Our finding that 61 of 147 (41%) of lung adenocarcinomas are ATM negative suggests that combinations of an ATR kinase inhibitor with standard-of-care cisplatin may improve clinical outcome in 100,000's of patients with ATM-deficient lung adenocarcinoma every year. A multicenter phase 1/phase 1b clinical trial with AZD6738 (NCT02264678) is currently recruiting patients in the United States, France, South Korea and the United Kingdom to test this hypothesis [38, 39].

## MATERIALS AND METHODS

### Patients and tissue microarray (TMA)

Samples were obtained by selection of lung cancer patients who underwent thoracic surgical procedures at the University of Pittsburgh Cancer Institute (2004–2011) and whose tumor tissues were stored as FFPE tissue in an IRB-approved tissue bank protocol. Tissue microarrays were constructed using randomly selected archival lung adenocarcinomas with known EGFR and KRAS mutation status. Hematoxylin- and eosin- stained sections were used to select areas of each FFPE tissue specimen that contained representative tumor cells. At least 4 cores of 1.0 mm in diameter were extracted from the selected area of each tissue block and arrayed on new recipient blocks using a commercially available microarray instrument (Beecher Instruments Manual Arrayer; Beecher Instruments, Inc., San Prairie, WI). Seven TMA blocks were generated which included 230 samples from 227 patients.

Clinical and pathological information for patients and tumors were abstracted from the UPCI cancer registry, supplemented by chart review: age, gender, race, smoking history, pathological stage, surgical procedure, adjuvant treatment, inflammation, angiolymphatic or pleural invasion, and overall survival from diagnosis.

### Detailed immunohistochemistry staining procedure

Rabbit monoclonal anti-ATM antibody [Y170] ab32420 was generated by Epitomics, who were subsequently purchased by Abcam, using a synthetic peptide around serine-1981.

1. Sections were de-waxed in two changes of xylene for 5 min each.
2. Sections were re-hydrated through graded alcohols (100%, 90% and 70%) for 5 min each.
3. Sections were washed in running water for 5 min.
4. Antigen retrieval was performed in a conventional pressure cooker in pH9 antigen retrieval buffer (Dako S2367) for 5 min at pressure.
5. Slides were rinsed in TBS-Tween 0.05% (TBS-T)
6. Endogenous peroxidase was blocked in 3% H2O2 in TBS-T for 10 min.
7. Slides were washed twice in TBS
8. Dako serum free protein block (X0909) was applied for 15 min.
9. Serum free protein block was blown off the slides (do not wash).
10. Sections were incubated with primary anti-ATM ab32420 antibody at 1.2 ug/ml in TBS-T for 60 min.
11. Slides were washed in TBS-T.
12. Sections were incubated with secondary antibody Dako ChemMate Envision (K5007) for 30 min.
13. Slides were washed twice in TBS-T.
14. Sections were incubated with chromagen Dako DAB peroxidase substrate solution (K5007) for 10 min.
15. Slides were washed in running water.
16. Sections were counterstained with Carazzi's haematoxylin.
17. Sections were dehydrated through graded alcohols (70%, 90% and 100%) for 5 min each.
18. Sections were cleared in two changes of xylene for 5 min each.
19. Slides were mounted using xylene based mounting media.

### Tissue culture

NCI-H460 were purchased from the American Type Culture Collection (ATCC) in November, 2013. Cells were periodically tested for mycoplasma (Lonza MycoAlert Mycoplasma Detection Kit). Experiments were conducted on cells with fewer than 20 passages after initial resuscitation. The shRNA targeting ATM has been described previously [27]. Cells were cultured in RPMI-1640 (containing 2 mM l-glutamine) supplemented with 10% FBS, penicillin/streptomycin in a humidified incubator at 37°C with 5% CO_2_. 293T cells were cultured in DMEM supplemented with 10% FBS, penicillin/streptomycin in a humidified incubator at 37°C with 5% CO_2_. 293T cells were transfected using Lipofectamine 2000 according to the manufacturer's instructions (ThermoFisher Scientific).

### Immunoblotting

Whole cell extracts were prepared in: 50 mM Tris-HCl pH 7.5, 150 mM NaCl, 50 mM NaF, 1% Tween-20, 0.5% NP40 and 1 × protease inhibitor mixture (Roche Applied Science, Indianapolis, IN).

ATM kinase inhibitors KU55933 (AstraZeneca, Macclesfield, UK) was used at final concentrations of 10 μM [28]. Cells were γ-irradiated in a Shepherd Mark I Model 68 [^137^Cs] irradiator (J.L. Shepherd & Associates, San Fernando, CA) at a dose rate of 71.1 Rad/min. SDS-PAGE using 3 – 8% Tris-Acetate gels (NuPAGE Novex) and Western blotting were performed using standard techniques. Anti-Flag was used at 1:1,000 (M5, Sigma-Aldrich) and anti-ATM ab32420 was used at 1:2,000.

### Statistical analysis

Overall survival (OS) was measured from the date of diagnosis to the date of death, or censored at the last date the patient was known to be alive. The primary analysis used Kaplan-Meier methods and the log-rank test to assess the univariate relationship between ATM status and OS. Multivariable Cox proportional hazards models were fitted to evaluate ATM in addition to known prognostic factors such as age, sex, and pathologic stage. For patients with multicentric disease, pathology could be updated from cancer registry entries to reflect the highest stage for synchronous disease. This reflected the use of pathology stage as a covariate reflecting the patient's prognosis rather than an independent marker for the tissue present in the TMA. Sample size was determined by the available tissue and follow-up events in the TMA, which was assembled for use by multiple University of Pittsburgh lung SPORE projects. Statistical analyses were conducted using SAS/STAT software, version 9.4 (SAS Institute, Inc., Cary, NC), with all p-values two-sided.

## SUPPLEMENTARY FIGURES


