# Supplementary material for: ATM protein is deficient in over 40% of lung adenocarcinomas
Source: Oncotarget. 2016 Jun 1;7(36):57714–25. doi: 10.18632/oncotarget.9757 (PMC5295384; doi:10.18632/oncotarget.9757)
Supplement: Supplementary file 1 [file oncotarget-07-57714-s001.pdf]

SUPPLEMENTARY FIGURES

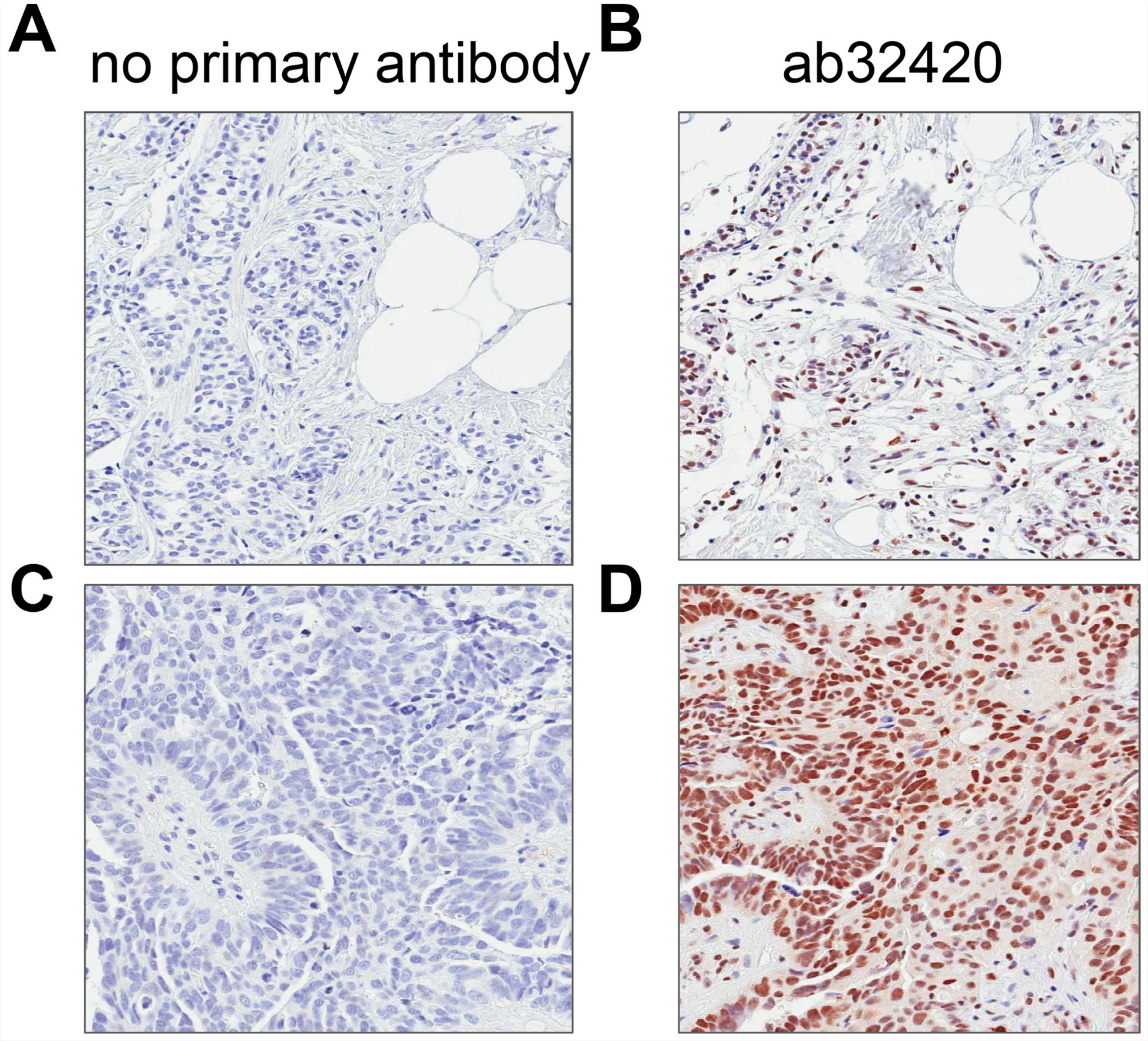

Supplementary Figure S1: No primary antibody controls are negative for staining. Sections were incubated in **A/C.** TBS-Tween 0.05% in place of **B/D.** primary antibody anti-ATM ab32420. Staining with the secondary antibody and detection reagents alone is neglible.

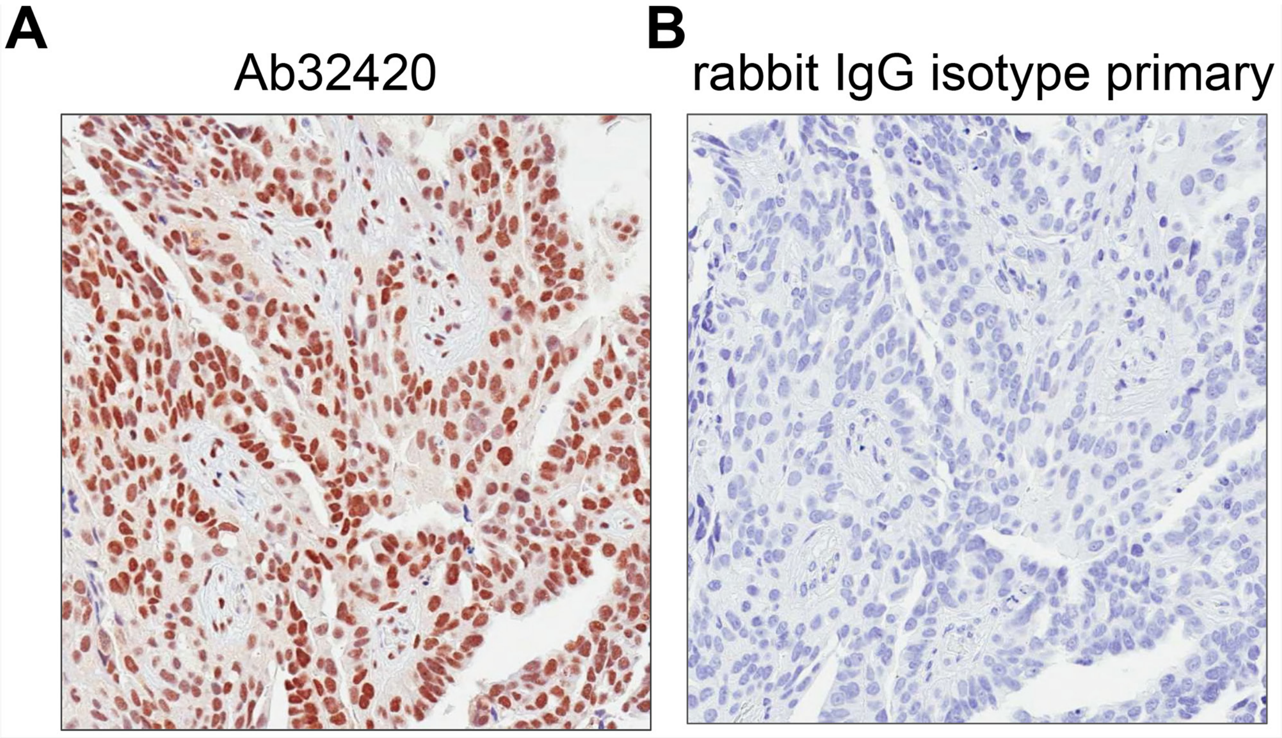

Supplementary Figure S2: Isotype control is negative for staining. Anti-ATM ab32420 **A.** was replaced with **B.** rabbit IgG (Dako X0936) isotype control primary control. Rabbit IgG is supplied at 5g/L. The concentration was adjusted to 1000 mg/ml in TBS-Tween 0.05%. Rabbit IgG was further diluted in TBS-Tween 0.05% to the same concentration as ab32420 (1000/1.22 giving a working concentration of 1:819).
